# A complete linkage disequilibrium in a haplotype of three SNPs in Fat Mass and Obesity associated (FTO) gene was strongly associated with anthropometric indices after controlling for calorie intake and physical activity

**DOI:** 10.1186/s12881-018-0664-z

**Published:** 2018-08-20

**Authors:** Naser Kalantari, Nastaran Keshavarz Mohammadi, Pantea Izadi, Maryam Gholamalizadeh, Saeid Doaei, Hassan Eini-Zinab, Tuire Salonurmi, Shahram Rafieifar, Reza Janipoor, Ghasem Azizi Tabesh

**Affiliations:** 1 Department of community nutrition, School of Nutrition and Food Sciences, Shahid Beheshti University of Medical Sciences, Tehran, Iran; 2 Department of public health, Shahid Beheshti University of Medical Sciences, Tehran, Iran; 3 Department of Medical Genetics, School of Medicine, Tehran University of Medical Sciences, Tehran, Iran; 4 Student Research Committee, Cancer Research Center, Shahid Beheshti University of Medical Sciences, Tehran, Iran; 5 Natural Products and Medicinal Plants Research Center, North Khorasan University of Medical Sciences, Bojnurd, Iran; 6 Department of public health, School of Public Health, North Khorasan University of Medical Sciences, Bojnurd, Iran; 7 Cancer Research Center, Shahid Beheshti University of Medical Sciences, Tehran, Iran; 8 Research groups of Internal Medicine, Faculty of Medicine, University of Oulu, Oulu, Finland; 9 Health Promotion and Education Department, Ministry of health, Tehran, Iran; 10 Department of Veterinary Medicine, School of Veterinary Medicine, Shiraz University of medical sciences, Shiraz, Iran; 11 Department of Medical Genetics, Faculty of Human Genetics, Tehran University of Medical Sciences, Tehran, Iran

**Keywords:** Fat mass and obesity associated (FTO), SNPs, Anthropometry, Haplotypes

## Abstract

**Background:**

The underlying mechanism of the effect of FTO genotype on body mass index (BMI) and body composition is unknown. The objective of the study was to investigate the association of FTO gene polymorphisms with anthropometric indices in adolescent boys after adjustments for dietary intake and physical activity.

**Methods:**

In this school-based study, we enrolled 123 male adolescents without extra weight and 110 male adolescents with body mass index (BMI) higher than + 1 Z-score. The DNA samples were genotyped for the FTO gene polymorphisms by DNA Sequencing. BMI and body composition were assessed using bioelectrical impedance analyzer scale. Association of the FTO polymorphisms with Weight, height, BMI, body fat percent and skeletal muscle percent were investigated. Data on potential confounders (calorie intake and physical activity) were collected through the use of pre-tested questionnaires.

**Results:**

Adolescents with higher BMI and body fat percent and lower skeletal muscle percent were more likely to have a newly found haplotype of rs9930506, rs9930501 & rs9932754 (GGT) in the first intron of the FTO with complete linkage disequilibrium (LD) compared with those with the lower BMI (6.15;2.28–16.63), body fat percent (9.54;0.92–47.44) and higher skeletal muscle percent (9.26;1.85–46.38). This association was not changed after controlling for age. Additional adjustments for calorie intake and physical activity did not alter the association.

**Conclusions:**

A haplotype in the first intron of the FTO gene had a strong association with obesity indices in adolescent boys after adjustments for calorie intake and physical activity. It’s suggested that the FTO genotype exert its effects on adolescents’ anthropometric indices as haplotype and through mechanisms other than changes in calorie intake and expenditure.

**Trial registration:**

This paper reports the first phase of a comprehensive interventional study (Interactions of Genetics, lifestyle and anthropometrics study or IGLA study) and is retrospectively registered in the Iranian Registry of Clinical Trials as IRCT2016020925699N2. Date registered: April 24, 2016. (http://www.irct.ir/searchresult.php?id=25699&number=2).

**Electronic supplementary material:**

The online version of this article (10.1186/s12881-018-0664-z) contains supplementary material, which is available to authorized users.

## Background

The relationship between FTO and obesity is reported by several studies [1–11]. Still there is no general agreement on the underlying mechanisms of effects of FTO on body weight. Recent studies showed that polymorphisms of FTO play key role on control of food intake. People with the AA or AT genotypes of rs9939609 polymorphism had significantly higher calorie intake than people with the TT genotype [12]. Moreover, obesity risk allele of FTO was associated with the less level of lipolysis in adipocytes that it shows possible primary role of the FTO gene in adipose tissue [13]. Many polymorphisms of the FTO gene are studied for the possible association with obesity. Some of these SNPs are rs178117449, rs9939609, rs3751812, rs1421085, rs9930506, and rs7202116 [14] and the recent studies have shown that the genotype of the rs9930506 polymorphism has the strongest influence on body weight and body composition [14, 15].

Although the aforementioned studies provide compelling evidence that the FTO gene is involved in determining anthropometric indices [12–15], the underlying mechanisms of the polymorphisms effect and whether the rs9930506 polymorphism has correlation with other SNPs or not is not clear. Hence, this study used DNA sequencing method to assess the genotype of the rs9930506 polymorphism and two other adjacent polymorphisms (rs9930501 & rs9932754) in the first intron of the FTO gene. This study aimed to investigation of the association between these SNPs and anthropometric indices (i.e. weight, height, BMI, body fat percent and skeletal muscle percent) after adjustments for dietary intake and physical activity.

## Methods

The following details are presented in accordance with the STROBE guidelines for cross-sectional studies.

### Study population

This paper reports the first phase of a comprehensive interventional study (Interactions of Genetics, lifestyle and anthropometrics study or IGLA study) and is registered in the Iranian Registry of Clinical Trials as IRCT2016020925699N2. The first phase of this study was carried out on adolescent male students of two schools from one district of Tehran over one school year from January to June 2016. The inclusion criteria were age 12 to 16 years, students’ willingness to participate in the study, and reaching the puberty stage (140 students of each schools). Stage of puberty was assessed by a pediatric psychologist using Tanner criteria of genital and pubic hair stage. The students who were suffering from diseases effective on body weight (*n* = 20), treated with the drugs that effect on body weight (*n* = 14), fear of blood sampling (*n* = 5), implausible data on BMI (*n* = 4) or difficulty in finding the veins (n = 4) were excluded. Finally, a total of 233 adolescent boys were studied (Additional file 1).

### Anthropometric assessments

A.ll students were assessed in terms of anthropometric parameters (i.e. weight, height, BMI, body fat percent (BF), and skeletal muscle percent (SM)). Heights were measured in the standing position and without shoes using a standard measuring tape attached to the wall. Students’ weights were measured and the values ​​of BMI, BF and SM were determined using a validated bioelectrical impedance analysis scale (Omron company model BF-511) [16] after entering their age, gender and height. The extracted data was classified according to World Health Organization z scores (for height, weight and BMI) and the published criteria reported by the recent studies (for BF and BM) [17]. Moreover, maturity situation of the students was examined by a psychologist.

### Genotyping

AS the FTO gene is large encompassing > 410 kb of genomic region [18], a targeted approach was adopted to search for common SNPs within 300 bp upstream and 300 bp downstream of the original and most significantly associated SNP, rs9930506 reported by Sentinelli et al. [19]. Five ml of blood samples were collected in EDTA tubes from each participants and were transported to the cellular and molecular laboratory at nutritional sciences and food technology school of Shahid Beheshti university of medical sciences to isolation of buffy coat, DNA extraction, and PCR.

DNA extraction kit of GeneAll Company was used to extract and purify the DNA sample. The NanoDrop device (Thermo Scientific, Wilmington, DE, USA) was used to quantify the DNA concentration. The Optical Density (OD) of samples were obtained in the absorption rate of 260 to 280 and confirmed if it was from 1.8 to 2. Moreover, to check the quality of extracted DNA, electrophoresis on agarose gel technique was used. After primer design (Additional file 2), genomic DNAs were amplified by PCR by use of Taq DNA Pol 2X Master Mix Red (cat. No A180301; Ampliqon, Denmark). The PCR product were sent to Geneall company for DNA sequencing. The quality and average length of sequence library for each sample was assessed using Chromas software (version 2.33, http://www.technelysium.com.au/chromas.html). The population were tested to determine whether the genotypes were in Hardy-Weinberg equilibrium by comparing the observed genotype frequencies in AAA cases and controls with their expected frequencies.

### Assessment of other variables

Usual dietary intakes of participants were examined by a validated 168-item semi-quantitative FFQ [20]. The FFQ consisted of 168 food items with standard portion sizes commonly consumed by Iranian people. A trained interviewer administered the FFQ through face-to-face interviews. All reported consumption frequencies were converted to grams per day by using household measures. Daily intakes of energy were measured for each person by using the modified US Department of Agriculture food consumption database, which was modified for Iranian foods.

The International Physical Activity Questionnaire (IPAQ) was used for measuring physical activity of participants through the face-to-face interview [21]. All results of the IPAQ were expressed as metabolic equivalents per minute (MET-minutes per week).

### Statistical analyses

Participants were categorized based on BMI in: no overweight, overweight (+ 1 z score), or obese (+ 2 z score). General characteristics and dietary intakes of study participants across categories of BMI were examined using one-way ANOVA for continuous variables and Chi square for categorical variables. The association of SNPs with quantitative anthropometric measures was assessed by using multinomial logistic regression in two models. Age (years) was adjusted in the first model. Additional controlling for calorie intake (kcal/day) physical activity (METs/week) was done in the second model. The statistical analyses were carried out by using SPSS version 23. *P* values were considered significant at < 0.05.

## Results

Students’ age and weigh mean were about 14 years (Table 1) and all of the students had passed the sexual maturity stage. Students with higher BMI had more calorie intake, lower physical activity, and shorter height, but these differences were not statistically significant. Moreover, overweight and obese students had significantly higher BF and lower SM compared to subjects without overweight.

**Table 1:** General characteristics of cases and controls

	No overweight (*n* = 122)	Overweight (*n* = 62)	Obese (*n* = 48)	*P* value
Age (years)	14.2 ± 0.9	13.8 ± 1.1	14.4 ±2.2	0.12
Physical activity (MET-minutes per week)	1218 ± 168.7	1006 ± 71.8	1004 ± 85.6	0.78
Calorie intake (kcal)	2388 ± 869.6	2399 ± 854.9	2475 ± 858.3	0.08
BMI (kg/m^2^)	18.8 ± 2.1	23.91.7	29.8 ± 3.1	< 0.001
FM (%)	13.6 ± 4.9	23.2 ± 6.1	31.1 ± 5.3	< 0.001
SM (%)	39.9 ± 2.8	36.9 ± 2.3	33.5 ± 2.7	< 0.001
Weight (kg)	51.1 ± 10.2	68.1 ± 10.8	85.7 ± 12.7	< 0.001
Height (cm)	176 ± 13.8	169 ± 9.1	167 ± 16.5	0.83
GG genotype (%)	18.9	45.9	35.2	< 0.001

Comparisons were made using ANOVA

### The FTO genotype

The A and G allele frequencies for these polymorphisms were 0.6 and 0.4, respectively, and the genotypes were in Hardy–Weinberg equilibrium. A complete LD in a haplotype block composed of 3 SNPs (rs993275 (allele G), rs9930506 (allele G), rs9930501 (allele C)) encompassing 40 bp in all students was identified. The rs930506 GG genotype was more frequent in overweight and obese subjects than subjects without overweight. Distribution of risk genotype of FTO according to categories of BMI are presented in Table 2.

**Table 2:** Distribution of the risk genotype of FTO polymorphisms across different categories of BMI

	No overweight (n = 122)	Overweight (n = 62)	Obese (n = 48)	*P* value
The rs9930506 GG genotype (%)	18.9	45.9	35.2	< 0.001
The rs993275 GG genotype (%)	18.9	45.9	35.2	< 0.001
The rs9930501 CC genotype (%)	18.9	45.9	35.2	< 0.001

### Association between the FTO haplotype and anthropometric indices

Multivariable-adjusted odds ratios and 95% confidence intervals for risk genotype of FTO across categories of BMI are shown in Table 3. Obese subjects were more likely to have risk genotype of FTO compared with those without overweight (5.87; 2.16–15.99). This association was not changed after controlling for age. Additional adjustments for calorie intake and physical activity did not alter the association (6.06; 2.19–16.58).

**Table 3:** Multivariable-adjusted odds ratios for the risk genotype of FTO across categories of BMI

	No overweight (n = 122)	Overweight (n = 62)	Obese (n = 48)	*P* value
Crude	1	6.26 (2.43–16.11)	6.15 (2.28–16.63)	< 0.001
Model 1	1	6.24 (2.42–16.1)	5.87 (2.16–15.99)	< 0.001
Model 2	1	6.35 (2.44–16.51)	6.03 (2.19–16.58)	< 0.001

Model1: adjusted for age

Model2: additionally, adjusted for calorie intake and physical activity

Multivariable-adjusted odds ratios and 95% confidence intervals for risk genotype of FTO across categories of BF are shown in Table 4. Higher BF was positively associated with odds of risk genotype of FTO (9.54; 0.92–47.44). This positive association did not alter even after controlling for age (9.12; 1.83–45.5). Even after controlling for confounders, including dietary intakes and physical activity, the association remained significant (9.26; 1.85–46.38).

**Table 4:** Multivariable-adjusted odds ratios for the risk genotype of FTO across categories of BF

	Low BF (BF < percentile 2)	Normal BF (percentile 2 < BF < percentile 91)	High BF (percentile 91 < BF < percentile 98)	Very high BF (BF > percentile 98)	*P* value
Crude	1	3.23 (0.72–14.52)	8.40 (1.53–46.23)	9.54 (0.92–47.44)	0.004
Model 1	1	3.01 (0.66–13.65)	7.87 (1.42–43.6)	9.12 (1.83–45.5)	0.005
Model 2	1	3.07 (0.07–1.5)	7.87 (1.41–44.01)	9.26 (1.85–46.38)	0.006

Model1: adjusted for age

Model2: additionally, adjusted for calorie intake and physical activity

Multivariable-adjusted odds ratios and 95% confidence intervals for risk genotype of FTO across categories of SM are shown in Table 5. Higher SM was negatively associated with odds of risk genotype of FTO (0.28; 0.12–0.62). This association was not changed after controlling for age (0.26; 0.11–0.59). Additional adjustments for calorie intake and physical activity did not significantly alter the association. (0.26;0.11–0.59).

**Table 5:** Multivariable-adjusted odds ratios for the risk genotype of FTO across categories of SM

	Low or normal SM (SM < percentile 98)	High SM (SM ≥ percentile 98)	*P* value
Crude	1	0.28 (0.12–0.62)	0.001
Model 1	1	0.26 (0.11–0.59)	0.001
Model 2	1	0.26 (0.11–0.59)	0.001

Model1: adjusted for age

Model2: additionally adjusted for calorie intake and physical activity

## Discussion

The results of the study identified that male adolescents with overweight, higher BF and lower SM were more likely to have the risk genotype of FTO haplotype compared with those without overweight. This association remained statistically significant even after controlling for common confounders, including calorie intake and physical activity. This study is the first to examine the association between a newly found FTO haplotype and anthropometric indices in adolescents.

The relationship between rs9930506 polymorphism and BMI has been studied frequently and the GG genotype of rs9930506 polymorphism was positively correlated with BMI. For example, in a study by Scuteri et al. to evaluate the polymorphisms associated with obesity, the results showed that the rs9930506 polymorphism had the strongest correlation with BMI [22]. Also, in the study conducted by Sentinelli et al. to determine the effects of rs9930506 polymorphism on BMI, the results showed that both polymorphisms were associated with higher BMI. In addition, rs9930506 was correlated with early onset of childhood obesity [15]. Although some studies have reported contradictory results [23–25]. Li et al. studied on the association between three polymorphisms of the FTO gene (rs8050136, rs9939609, and rs9930506) with obesity, the results showed that none of the studied SNPs are associated with obesity and waist circumference [23]. Moreover, Wang et al. studied the relationship between FTO polymorphisms (including rs9930506) and metabolic syndrome and the rs9930506 polymorphism did not show a significant relationship with the pathogenesis of metabolic syndrome including abdominal obesity [25]. However, both of these negative studies were conducted on the Chinese people. The MAF of this polymorphism in Chinese communities is significantly less than that in European populations [22].

Two other SNPs (rs9930501 & rs9932754) have been less studied for their potential impact on anthropometric measurements [26, 27]. The association between rs9932754 and BMI and its same MAF with rs9930506 was previously reported [27]. In the present study a complete LD was interestingly found between three neighboring SNPs of intron 1 region of the FTO gene. Although the linkage disequilibrium (LD) between rs9930506, rs9930501 and rs9932754 has been indirectly mentioned in genome-wide association studies [24, 28, 29]. The results of the present study suggested that the FTO gene polymorphisms play their roles as a haplotype (and not single SNPs). Given the close proximity of these polymorphisms, it can be also considered as a new hypothesis that these polymorphisms exert their effects on expression of the other genes through a sequence of intron 1 region. Recent studies suggested that rather than affecting FTO itself, FTO polymorphisms in this region influence expression of a distant gene, IRX3. It’s suggested that this non-coding region of FTO gene interacts with the IRX3 promoter and changes its expression level [30].

This study has some limitations as well. We used self-reported data on dietary intake and physical activity. It was frequently reported that obese persons underestimate their food intake and overestimate their physical activities. Moreover, we enrolled only male adolescents mainly due to gender segregation in schools. The association between FTO genotype and anthropometric measurements may be affected by gender.

## Conclusion

The results of this study showed that a haplotype in the first intron of the FTO gene had a strong association with obesity indices in Iranian adolescent boys after adjustments for calorie intake and physical activity. It seems that the effects of FTO genotype on anthropometric indices is independent from energy intake and energy expenditure. Future studies in this field are warranted to identify the underlying mechanisms to gain a better understanding into the interactions between the FTO gene polymorphisms and anthropometric measurements.

## Additional files

Additional file 1: Participant Flow Diagram, Study population diagram (DOC 12 kb)

Additional file 2: Sequences of primers used in this study, Sequences of primers used for sequencing (DOCX 26 kb)

## Abbreviations

BF: Body fat percent
BMI: Body mass index
FTO: Fat mass and obesity-associated
GWAS: A genome-wide association study
LD: Linkage disequilibrium
SM: Skeletal muscle percent
SNPs: Single-nucleotide polymorphisms
