# Supplementary material for: A complete linkage disequilibrium in a haplotype of three SNPs in Fat Mass and Obesity associated (FTO) gene was strongly associated with anthropometric indices after controlling for calorie intake and physical activity
Source: BMC Med Genet. 2018 Aug 20;19:146. doi: 10.1186/s12881-018-0664-z (PMC6102807; doi:10.1186/s12881-018-0664-z)
Supplement: Supplementary file 1 — Participant Flow Diagram, Study population diagram (DOC 12 kb) [file 12881_2018_664_MOESM1_ESM.doc]

**Additional file 1: Participant Flow Diagram**

Analysed (n= 237)
♦ Excluded from analysis (n=0)

## Analysis

## Enrollment

Excluded (n=47)

♦  Not meeting inclusion criteria (n=34)

♦  Declined to participate (n=5)

♦  Other reasons (n=8)

Assessed for eligibility (n=280)
