# Supplementary material for: A complete linkage disequilibrium in a haplotype of three SNPs in Fat Mass and Obesity associated (FTO) gene was strongly associated with anthropometric indices after controlling for calorie intake and physical activity
Source: BMC Med Genet. 2018 Aug 20;19:146. doi: 10.1186/s12881-018-0664-z (PMC6102807; doi:10.1186/s12881-018-0664-z)
Supplement: Supplementary file 2 — Sequences of primers used in this study, Sequences of primers used for sequencing (DOCX 26 kb) [file 12881_2018_664_MOESM2_ESM.docx]

Additional file 2: Sequences of primers used in this study.

| **Intron** |  | **Primer sequences (5′-3′)** | **PCR fragment length (bp)** |
| --- | --- | --- | --- |
| 1 | F | CAA AGG TGG GCA TAG AGA TTG | 371 |
| 1 | R | ACG TGC CTA TAA AAC TGG GC |  |
